# Misfolding of a DNAzyme for ultrahigh sodium selectivity over potassium

**DOI:** 10.1093/nar/gky807

**Published:** 2018-09-12

**Authors:** Yanping He, Da Chen, Po-Jung Jimmy Huang, Yibo Zhou, Lingzi Ma, Kexin Xu, Ronghua Yang, Juewen Liu

**Affiliations:** 1 State Key Laboratory of Precision Measurement Technology and Instruments, University of Tianjin, Tianjin 300072, China; 2 Department of Chemistry, Waterloo Institute for Nanotechnology, University of Waterloo, Waterloo, ON N2L 3G1, Canada; 3 School of Chemistry and Biological Engineering, Changsha University of Science and Technology, Changsha 410114, P. R. China

## Abstract

Herein, the excellent Na^+^ selectivity of a few RNA-cleaving DNAzymes was exploited, where Na^+^ can be around 3000-fold more effective than K^+^ for promoting catalysis. By using a double mutant based on the Ce13d DNAzyme, and by lowering the temperature, increased 2-aminopurine (2AP) fluorescence was observed with addition of both Na^+^ and K^+^. The fluorescence increase was similar for these two metals at below 10 mM, after which K^+^ took a different pathway. Since 2AP probes its local base stacking environment, K^+^ can be considered to induce misfolding. Binding of both Na^+^ and K^+^ was specific, since single base mutations could fully inhibit 2AP fluorescence for both metals. The binding thermodynamics was measured by temperature-dependent experiments revealing enthalpy-driven binding for both metals and less coordination sites compared to G-quadruplex DNA. Cleavage activity assays indicated a moderate cleavage activity with 10 mM K^+^, while further increase of K^+^ inhibited the activity, also supporting its misfolding of the DNAzyme. For comparison, a G-quadruplex DNA was also studied using the same system, where Na^+^ and K^+^ led to the same final state with only around 8-fold difference in *K*_d_. This study provides interesting insights into strategies for discriminating Na^+^ and K^+^.

## INTRODUCTION

Specific binding of metal ions is an interesting challenge. For example, Li^+^, Na^+^ and K^+^ are very similar to each other. With the same charge and preference for oxygen-based ligands, they mainly differ by size. In addition to relying on size and charge for metal recognition (e.g. by chemically designed ligands), biopolymers may harness additional mechanisms such as folding, catalysis and allosteric effects (1,2). Metal sensing has advanced significantly using DNA with catalytic activities known as DNAzymes (3–6). Many RNA-cleaving DNAzymes have been isolated requiring specific metals (7–10), such as Pb^2+^ (11), Zn^2+^ (12,13), Cu^2+^ (14), Cd^2+^ (15), UO_2_^2+^ (16), Hg^2+^ (17), Na^+^ (18–20), Ag^+^ (21) and lanthanides (22–24).

Recently, a few sodium-specific DNAzymes were reported with extremely high specificity (18–20,25–26). The NaA43 DNAzyme was reported by Lu *et al.*, and Na^+^ alone can activate it (18). Its analytical applications for Na^+^ detection were also demonstrated (18,27). We reported the Ce13d DNAzyme with a similar structure (22), but it requires a lanthanide ion in addition to Na^+^ (25). Their Na^+^ specificity is due to a common aptamer motif for Na^+^ (25,28–31). G-quadruplex DNA is often selectively stabilized by K^+^, but their selectivity for K^+^ over Na^+^ is typically below 50-fold (32–34). Since the Na^+^ selectivity of these DNAzymes is surprisingly high, it is important to rationalize such high selectivity. To study this, a sensitive probe for measuring metal binding is needed.

2-aminopurine (2AP) is a fluorescent adenine analog and its fluorescence is influenced by nearby base stacking and thus local folding of DNA (35–37). We recently optimized a highly sensitive 2AP-modified Ce13d mutant showing around 6-fold fluorescence increase upon Na^+^ binding at room temperature (28). Using this mutant, we herein aim to understand metal specificity and new strategies for metal ligand design.

## MATERIALS AND METHODS

### Chemicals

All of the DNA samples used in this work were from Integrated DNA Technologies (Coralville, IA, USA), and the sequences are listed in Supplementary Table S1. The buffers were from Mandel Scientific (Guelph, ON, Canada). The metal salts were from Sigma-Aldrich. Milli-Q water was used to prepare all the buffers and solutions. Our water, buffers and LiCl and KCl solutions were all analyzed by ICP-MS (ALS Environmental, Waterloo, ON) and their Na^+^ contents were all below the detection limit of the instrument.

### 2AP fluorescence spectroscopy

The DNAzyme complexes were annealed in buffer A (25 mM LiCl, 50 mM HEPES, pH 7.4) by heating the samples to 80°C for 2 min and then gradually cooling to 4°C over 30 min. Note the pH of the buffers was adjusted using LiOH to avoid Na^+^. When 2AP was modified on the substrate strand, the complex was prepared with 1 μM substrate and 2 μM enzyme. When the 2AP was on the enzyme strand, 2 μM substrate and 1 μM enzyme were used. The 2AP emission was measured immediately after adding a small volume of metal solutions in a 1 × 1 cm quartz fluorescence cuvette using a Cary Eclipse fluorometer by exciting at 310 nm. The spectra were recorded from 360 to 450 nm, and the peak intensity at 370 nm was used for quantification. The *K*_d_ value was calculated based on: *F* = *F*_0_ +*a*[M^+^]/(*K*_d_ +[M^+^]), where *F*_0_ and *F* are the fluorescence intensity before and after adding metal, respectively; [M^+^] is the metal concentration; and *a* is fitting constant. Most measurements in this work were carried out in triplicate and the standard deviations were plotted as the error bars.

### Activity assays

The DNAzyme complex was annealed in buffer B (25 mM NaCl, 50 mM MES, pH 6.0) with 10 μM FAM (carboxyfluorescein)-labeled substrate and 20 μM enzyme. After cooling to room temperature, the complex was diluted 10-fold in buffer C (25 mM LiCl, 50mM MOPS, pH 7.0) with a final of 1 μM substrate and 2 μM enzyme, and a metal solution was added to initiate the cleavage reaction. Note here we used MOPS buffer for the activity assay since we noticed some artifacts for gel running with HEPES. At each designated time point, the reactions were quenched by 8 M urea. The cleavage products were separated using 15% denaturing polyacrylamide gel electrophoresis (dPAGE) and analyzed by a ChemDoc MP imaging system (Bio-Rad). The data were fitted with the first-order rate equation *Y*_t_ = *Y*_0_ + *a*(1-*e*^−^*^kx^*), where *Y*_t_ and *Y*_0_ are cleavage fraction at time *t*, and at 0 min, respectively; and *k* is the observed rate constant.

### DMS footprinting

A 3′-end FAM labeled enzyme (Ce13d-FP-FAM) and its corresponding non-cleavable substrate were used. The DNAzyme complex ([Ce13d-FP-FAM] = 10 μM, [Sub-dA-FP] = 20 μM) was annealed in 50 mM MOPS buffer (pH 7.0) containing 25 mM LiCl. Then 600 mM various monovalent metal ions were respectively added. For methylation, 1.5 μl of freshly prepared 4% dimethyl sulfate (DMS) was added to 10 μl DNAzyme complex, followed by 15 min incubation in dark at room temperature, or 60 min incubation at 7°C. The reaction was then quenched by adding a solution (1 M β-mercaptoethanol, 600 mM NaOAc, pH 5.2, 200 μl), followed by isopropanol precipitation overnight at −20°C. The pellets were washed with 150 μl ethanol (70%) twice and suspended in 50 μl freshly prepared piperidine (10%). The DNA was cleaved at the methylated sites after heating at 90°C for 30 min, followed by vacuum drying at 60°C for 30 min to remove piperidine. The products were suspended in 8 M urea and analyzed by 15% dPAGE.

## RESULTS AND DISCUSSION

### Na^+^ selectivity from DNAzyme activity

**Figure 1.**
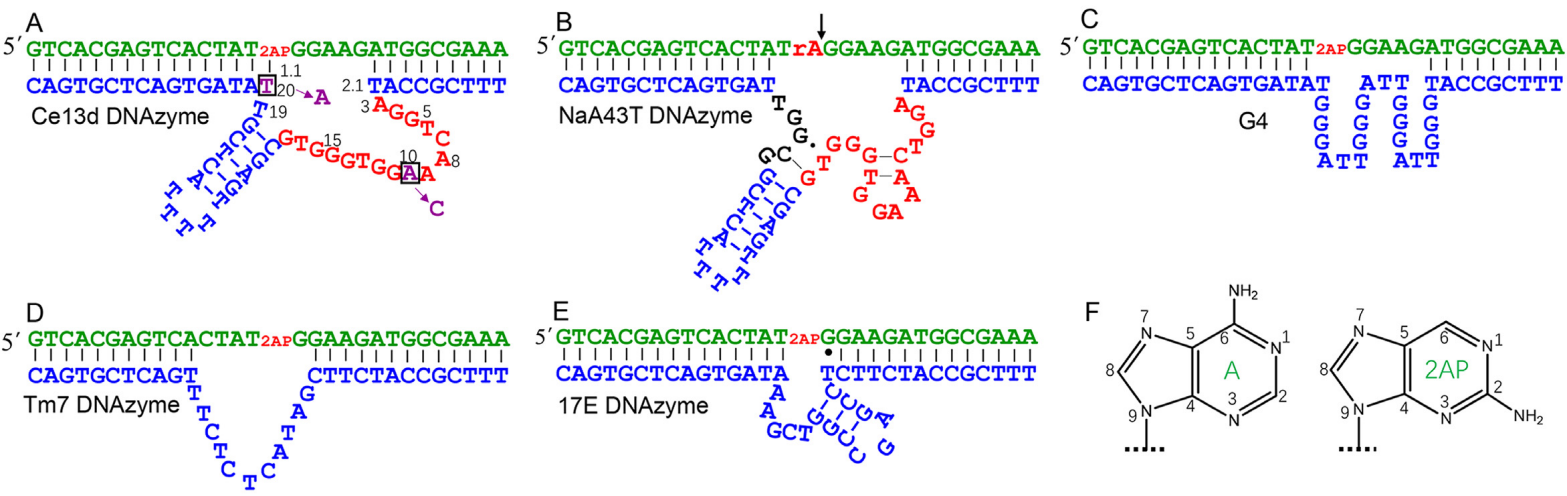
The secondary structures of the (**A**) Ce13d; (**B**) NaA43T; (**D**) Tm7; and (**E**) 17E DNAzymes. For some of them, both the cleavable rA containing substrate and the non-cleavable 2AP-modified substrate were tested. In (A), the A10C and T20A double mutations are shown and this double mutant is called Ce13p, which was used for most of the 2AP experiment. (**C**) The DNAzyme loop was replaced by a G-quadruplex structure. (**F**) The structures of adenine and 2AP.

The structure of the Ce13d DNAzyme is shown in Figure 1A. The enzyme strand has a hairpin and a large loop marked in red, and this loop is the main structure housing the Na^+^ aptamer (25,29–30). To measure the cleavage activity, a chimeric substrate with a single RNA linkage (rA or ribo-adenine) was used. Ce13d requires both Ce^3+^ and Na^+^ for cleavage activity (25). In addition to Ce13d, we also studied the NaA43 DNAzyme, which has the same Na^+^ binding motif. We shortened the literature reported NaA43 so that it mainly differs from Ce13d in the small segment (in black) close to the 3′ of the enzyme. This shortened version is named NaA43T here (Figure 1B), while the original sequence of NaA43 is shown in Supplementary Figure S1. NaA43 and NaA43T cleave its substrate in the presence of Na^+^ alone (18).

The NaA43T and Ce13d DNAzymes are both highly selective for Na^+^ (18,25). Our main focus of this study is the Na^+^ aptamer part, which is the same for both DNAzymes. In this work, we wanted to quantitatively measure metal specificity, and the NaA43T DNAzyme was chosen for the following reason. Ce13d needs 10 μM Ce^3+^ in addition to Na^+^ (25), but its activity was inhibited by a high concentration of Na^+^, e.g. 1 M Na^+^ (Supplementary Figure S2). This was likely due to weakened binding of Ce^3+^ by the DNAzyme with such a high salt concentration (38). To push for activity in the presence of other metals, namely Li^+^ and K^+^, high metal concentrations were tested and NaA43T can avoid this artefact and simplify data analysis.

**Figure 2.**
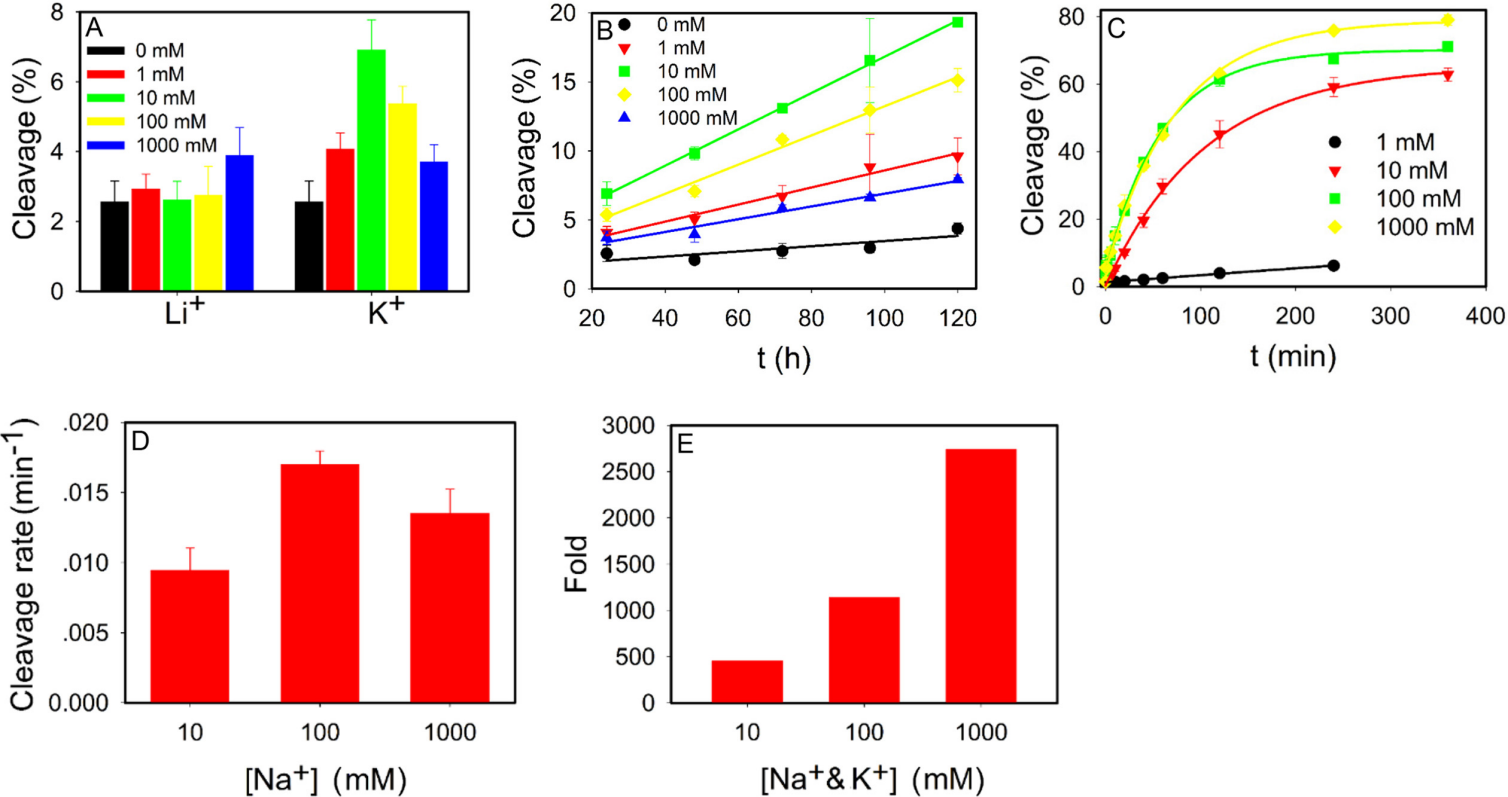
Cleavage activity assays using the NaA43T DNAzyme. (**A**) Cleavage yield after 24 h incubation with various concentrations of Li^+^ and K^+^. Kinetics of cleavage in the presence of various concentrations of (**B**) K^+^ and (**C**) Na^+^. (**D**) Cleavage rates with different concentrations of Na^+^. (**E**) The fold of cleavage rate difference of Na^+^ over K^+^. The reaction buffer was 50 mM MOPS, pH 7.0 with 25 mM Li^+^. The error bars represent the standard deviation from three independent measurements.

We incubated NaA43T with up to 1 M K^+^ or Li^+^ for 24 h. The Li^+^ samples did not show much cleavage compared to the background. Interestingly, after background subtraction, 4.3% cleavage was observed with 10 mM K^+^ (Figure 2A), whereas further increase of K^+^ gradually lowered the yield. To confirm this, we performed a kinetic measurement over 5 days (Figure 2B). A time-dependent cleavage with 10 mM K^+^ indeed occurred, and the rate dropped at higher K^+^ concentrations. ICP was performed to confirm that our Li^+^, K^+^ and buffers were free of Na^+^. Thus, the observed cleavage in K^+^ should not be due to Na^+^ contamination. In addition, the lower cleavage yield with 100 mM K^+^ than with 10 mM K^+^ also argued against Na^+^ contamination.

We then measured its cleavage kinetics in the presence of Na^+^ (Figure 2C), and the cleavage rates are plotted in Figure 2D. The rate saturated at around 100 mM Na^+^, and this was consistent with its previously reported apparent *K*_d_ of ∼40 mM Na^+^ (18). The cleavage rate with 10 mM K^+^ was estimated to be 0.0012 h^−1^, which was 454-fold slower than that with 10 mM Na^+^. If we compare the rate at 1 M metal, the difference reached nearly 3000-fold (Figure 2E). This improvement was mainly from the dropped activity of K^+^, which seemed to limit its own activity. Discrimination of Na^+^ and K^+^ was observed in other biological systems such as the Na^+^ channels, and discrimination is around 10- to 700-fold (39–41). It is intriguing that such high specificity was achieved by a simple DNA molecule.

### Local folding and misfolding

To measure metal binding, this rA was replaced by a deoxy-2AP nucleotide as shown in Figure 1A (28). 2AP is a fluorescent adenine analog (Figure 1F), and they have similar base pairing properties (37). The fluorescence of 2AP is highly dependent on its base stacking with neighboring nucleotides, and thus can reflect local folding of nucleic acids (36,42). Since 2AP only probes its nearby nucleotides, global structural changes cannot be concluded from 2AP-based experiments. In this work, folding is sometimes used in short for local folding.

To test if Na^+^ binding can quantitatively explain its excellent selectivity based on cleavage activity, we then used 2AP as a probe to measure metal binding. The original Ce13d DNAzyme produced only 2.5-fold fluorescence enhancement by Na^+^, and the increase by K^+^ and Li^+^ was even lower and cannot be accurately measured (Supplementary Figure S3). Similarly, we did not see much 2AP fluorescence change with the NaA43 DNAzyme (Supplementary Figure S4). For our purpose, a stronger signal increase was critical.

**Figure 3.**
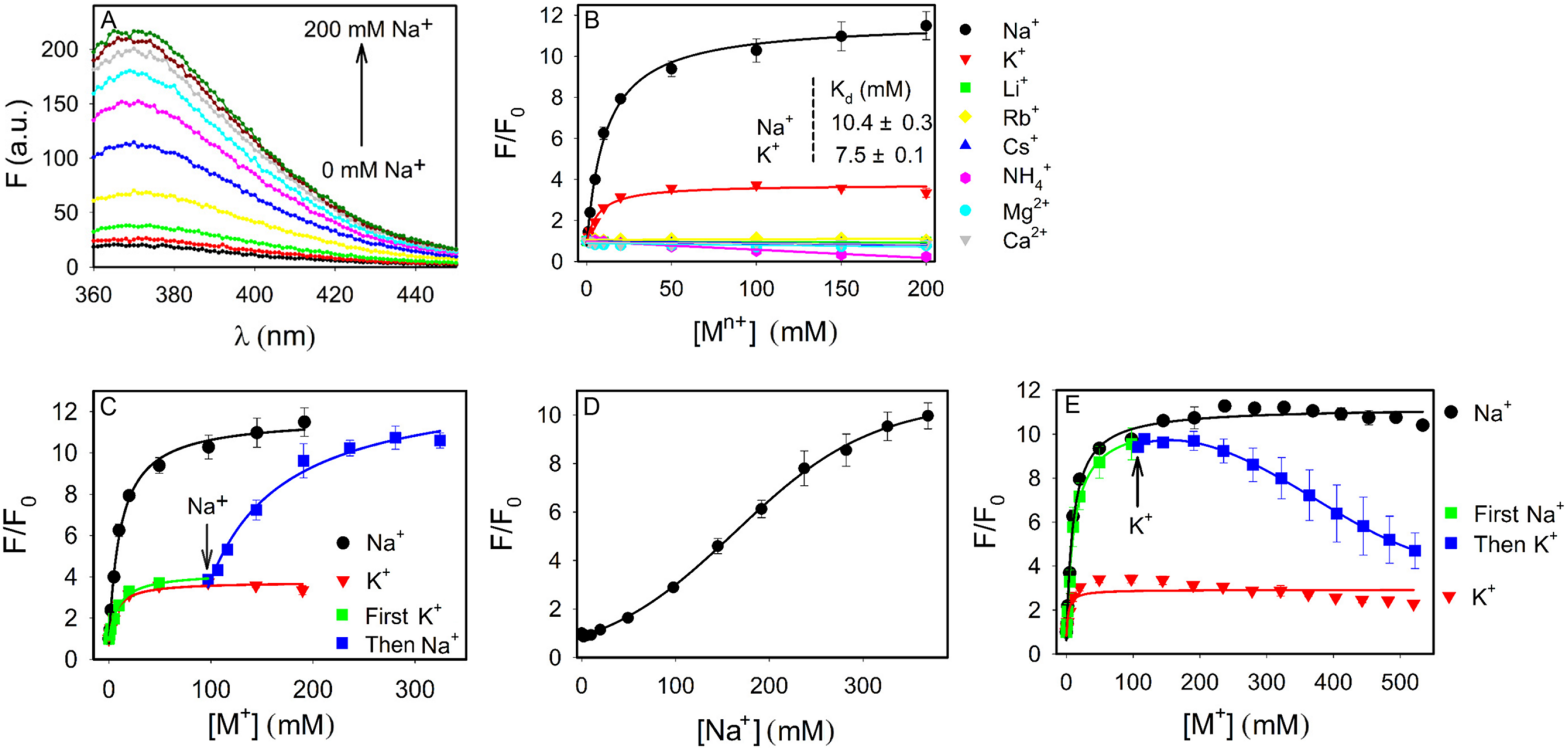
(**A**) Fluorescence spectra of the 2AP-labeled Ce13p DNAzyme with 310 nm excitation in the presence of various concentrations of Na^+^ in buffer (50 mM HEPES, pH 7.4, 25 mM LiCl) at 7°C. (**B**) Normalized 2AP fluorescence of Ce13p in the presence of various metal ions. (**C**) Titrating K^+^ first followed by Na^+^. (**D**) With 500 mM Li^+^ in buffer, the binding of Na^+^ was weaker. (**E**) Titrating Na^+^ first followed by K^+^.

Our previous work has produced an interesting double mutant with an excellent signal enhancement (28). The two positions of mutation are shown in Figure 1A: the adenine at 10 position (called A10) mutated to C, and T20 to A. This double mutant was named Ce13p. We followed the 2AP fluorescence spectra of the Ce13p DNAzyme as a function of Na^+^ concentration (Figure 3A). The 370 nm peak increased over 11-fold when Na^+^ was titrated to 200 mM at 7°C. At room temperature, we previously observed 6-fold increase. Thus, lowering temperature has produced an even higher signal change.

Using this probe, we titrated different metal ions (Figure 3B). While Na^+^ had the highest response, K^+^ also induced an obvious fluorescence increase. Other metals including monovalent (Li^+^, Rb^+^, Cs^+^ and NH_4_^+^), and divalent (Mg^2+^ and Ca^2+^) cations did not show any signal when titrated up to 200 mM. Therefore, this 2AP DNAzyme system was highly stable and non-specific metal interactions cannot change local folding near the 2AP. Interestingly, K^+^ and Na^+^ showed a very similar *K*_d_ of ∼10 mM (inset of Figure 3B), suggesting that both metals can bind to the DNAzyme. Although a clear difference can be observed for these two metals here, this difference was much less than that from the cleavage activity. A close examination of metal titration to the wild-type Ce13d DNAzyme also indicated a very small signal for K^+^, but not Li^+^ (Supplementary Figure S5). Therefore, both the wide-type Ce13d and this double mutant Ce13p have a similar trend for metal binding: Na^+^ giving the most folding followed by K^+^, while other metals showing no measurable binding.

The saturated fluorescence increase at 150 mM metal concentration was over 11-fold for Na^+^, while only 3.5-fold for K^+^. Since all these experiments started with the same initial state and 2AP fluorescence, the final states must be different for Na^+^ and K^+^. Therefore, even though the DNAzyme can bind K^+^, it has a different mode of binding compared to Na^+^. In other words, in terms of local folding, K^+^ can be considered to induce misfolding. This can explain the excellent Na^+^ selectivity from catalytic activity. Activation of the DNAzyme would require Na^+^ binding to reach an active state, while K^+^ misfolded the DNAzyme and thus inhibited its activity.

To further characterize metal binding, we first titrated 100 mM K^+^ and then added Na^+^ (Figure 3C). In this case, the fluorescence further increased. The *K*_d_ for the Na^+^ portion was 76.1 mM. Therefore, the DNAzyme can go from the K^+^ binding state to the Na^+^ binding state. The increased *K*_d_ could be due to K^+^ acting as a background electrolyte. To confirm this, we then turned our attention to Li^+^, which did not produce any change in the 2AP fluorescence. Therefore, Li^+^ can be considered as a background salt without specific interactions with the DNAzyme. We started with a high concentration of 500 mM Li^+^ and then added Na^+^ (Figure 3D). This background Li^+^ increased the *K*_d_ of Na^+^ binding to 169 mM, while the final fluorescence still reached over 10-fold increase. Therefore, Li^+^ non-specifically competed with Na^+^ but the same final binding state could still be reached. At last, we added Na^+^ first followed by K^+^ (Figure 3E). With 500 mM K^+^, the fluorescence dropped to around 4-fold, suggesting that the DNAzyme went from the Na^+^ binding state to the K^+^ binding state. Therefore, these two binding states can readily interconvert, suggesting a low activation energy barrier.

### A G-quadruplex DNA control

**Figure 4.**
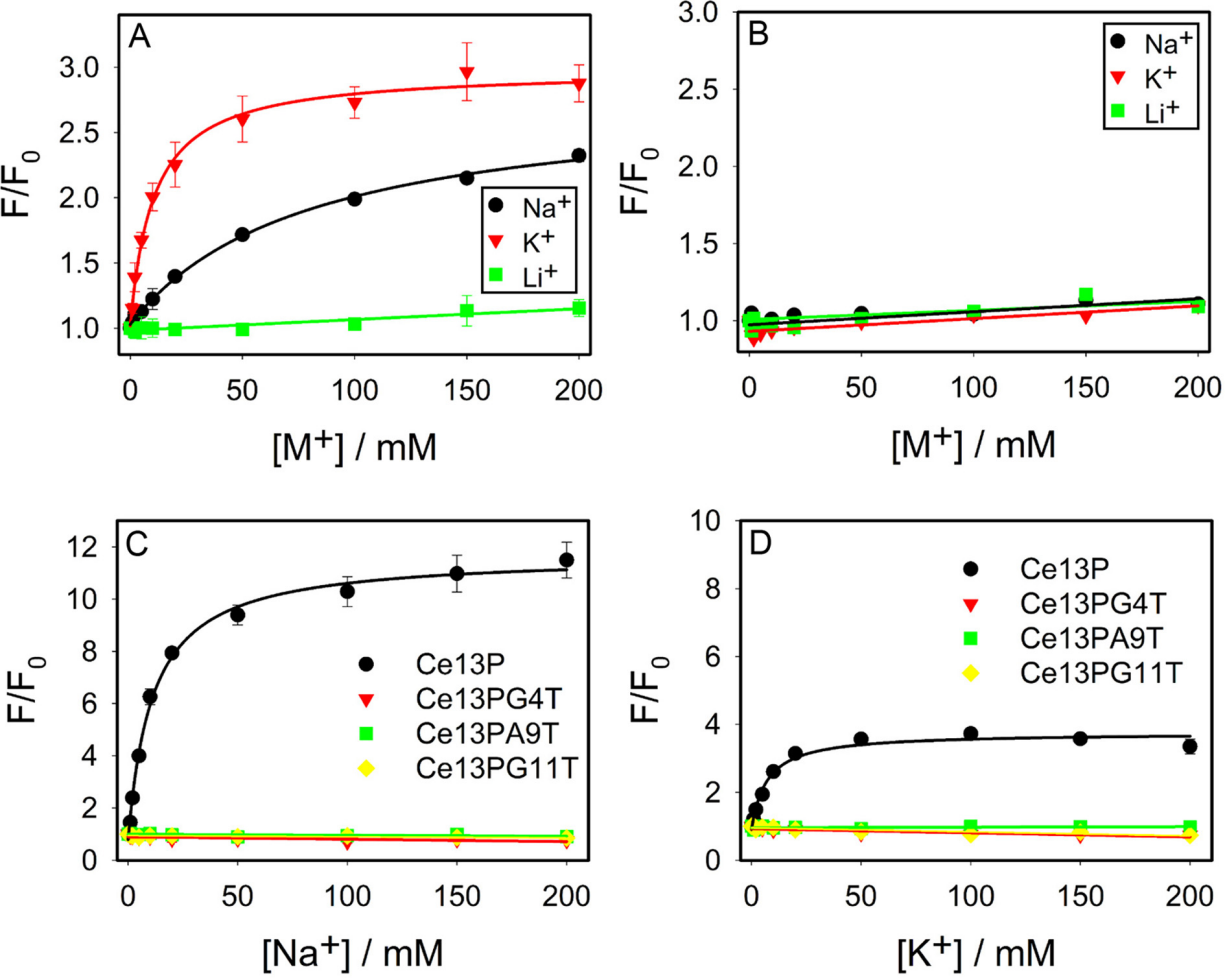
Titration of the three metals to (**A**) a G4 DNA sequence shown in Figure 1C, and (**B**) the Tm7 DNAzyme at 7°C in buffer (50 mM HEPES, pH 7.4, 25 mM LiCl). In both cases, the same 2AP-labeled substrate strand was used. Fluorescence enhancement of a few mutants based on the 2AP-modified Ce13p DNAzyme with different concentrations of (**C**) Na^+^ and (**D**) K^+^.

Specific binding of Na^+^ by DNA was studied only recently, whereas better known examples are G-quadruplex (G4) DNAs stabilized by K^+^ (43). Most G4 DNAs can be efficiently stabilized by K^+^, while to a lesser extent by Na^+^. Li^+^ in general cannot stabilize G4. Therefore, G4 DNA might be a good system for comparison. To maximally retain the similarity to our current system, we replaced the catalytic core of the DNAzyme by a G4 sequence (Figure 1C) (44). In this case, the same substrate strand with the 2AP label was used. Adding K^+^ enhanced the fluorescence by 2.7-fold with a *K*_d_ of 10.5 mM (Figure 4A). Na^+^ induced a slightly lower enhancement with a *K*_d_ of 80.3 mM, while Li^+^ did not change the fluorescence. This result is expected for a typical G4 DNA to discriminate K^+^ and Na^+^. From the Na^+^ fitted equation: *F* = 1.03 + 1.77[Na^+^]/(80.3+[Na^+^]), with an infinitely high Na^+^ concentration, its enhancement can also reach 2.8-fold, suggesting Na^+^ and K^+^ had the same final folding state. It just took more Na^+^ to fold. This example further confirmed that the final Na^+^ and K^+^ binding states were different for the DNAzyme.

We also studied another DNAzyme named Tm7, which is also lanthanide-dependent with a simple loop structure (Figure 1D). In this case, we did not observe folding with any of the metal (Figure 4B). This control also supported specific metal binding by Ce13p and the G4 sequence.

### Specific misfolding

So far, we demonstrated that both Na^+^ and K^+^ can bind to the Ce13p DNAzyme, but the way of binding appeared to be different. To further test binding specificity, we made point mutations to the DNAzyme and titrated them with Na^+^ (Figure 4C) and K^+^ (Figure 4D). Ce13p has quite a few guanine residues in the aptamer loop. We mutated certain guanines and also some non-guanine residues. It is interesting to note that all these single-nucleotide mutants fully inhibit fluorescence change in the presence of either Na^+^ or K^+^. Similar results were also obtained when we mutated the original Ce13d DNAzyme (Supplementary Figure S6). Therefore, binding was specific for both Na^+^ and K^+^ since a mutation in the enzyme loop fully inhibited metal binding. In other words, K^+^ binding was also specific. In reference to the cleavage activity, K^+^ induced local misfolding and this specific misfolding explains the high specificity for Na^+^.

### Reaction thermodynamics

**Figure 5.**
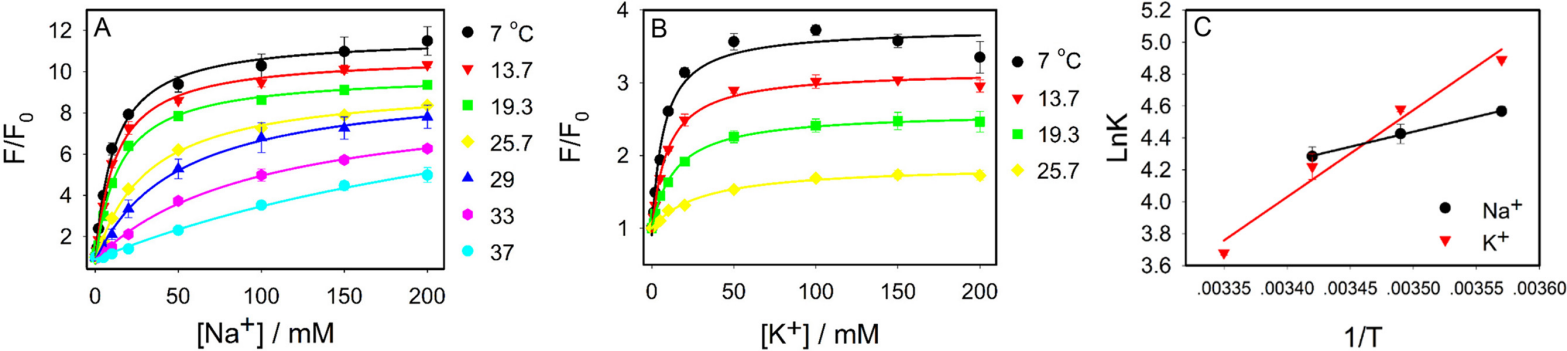
Temperature-dependent 2AP fluorescence change for the Ce13p DNAzyme by titrating (**A**) Na^+^ and (**B**) K^+^. (**C**) Van’t Hoff plot of the binding reactions to extract thermodynamic parameters.

To obtain further insights, we then measured the thermodynamics of Na^+^ and K^+^ binding. We titrated Na^+^ or K^+^ to the Ce13p DNAzyme at various temperatures (Figure 5A and B). At each temperature, the binding curves were fitted to obtain the *K*_d_ or *K*_a_ values, from which the Δ*G* of the reaction could be calculated. Na^+^ showed binding from 7°C to 37°C, but the data were far from saturation above 20°C and we only used the lower temperature data for the calculation. Similarly, binding of K^+^ disappeared beyond 25°C. These low temperature data were fitted to the van’t Hoff equation (Figure 5C), from which the Δ*H* and Δ*S* values were obtained (Table 1). For comparison, data were also cited from the literature for some G4 DNAs, for which UV-vis and CD spectroscopy were used. These techniques however did not give much signal change for our DNAzyme (data not shown), and we relied on fluorescence titration here.

**Table 1.** Thermodynamic parameters of the Ce13p DNAzyme and some literature reported G4 DNA binding to Na^+^ and K^+^

DNA	Cation	Δ*H*(kcal mol^−1^)	Δ*S*(cal mol^−1^K^−1^)	Δ*G* (310K)(kcal mol^−1^)	Ref.
Ce13p^a^	Na^+^	−3.7 ± 1.1	−4.3 ± 3.8	−2.4 ± 0.1	This work
Ce13p^b^	K^+^	−10.8 ± 0.8	−28.8 ± 2.6	−1.9 ± 0.0	This work
5′-(TTAGGG)_4_	Na^+^	−38	−119	−1.4	(56)
	K^+^	−49	−147	−3.8	(56)
5′-AGGG(TTAGGG)_3_	Na^+^	−54	−163	−3.1	(57)
	K^+^	−57	−169	−4.4	(57)
5′-TTAAGGG(TTAGGG)_3_	Na^+^	−38.5	−121	−0.9	(57)
	K^+^	−63	−193	−3.5	(57)
5′-GGG(TGTGGG)_3_	Na^+^	−45	−139	−1.9	(57)
	K^+^	−64	−189	−5.4	(57)

Note:^a^:7–19.3°C, ^b^:7–25.7°C.

In terms of binding affinity (reflected from Δ*G*), K^+^ was more favorable than Na^+^ for all the listed G4 DNA sequences; but for our DNAzyme, Na^+^ was slightly stronger than K^+^. Our Δ*G* values were lower than that for K^+^ binding to G4 DNAs. Similar to the G4 DNAs, our DNAzyme binding reactions were driven by enthalpy (entropy decreased). This is not surprising for cations binding to the nucleic acids with large electrostatic interactions. The absolute values of Δ*H* and Δ*S* in our DNAzyme system were, however, much lower (e.g. by over 10-fold for Na^+^ and a few fold for K^+^), suggesting that these metals did not fully interact with the DNAzyme. In G4 DNAs, K^+^ is fully dehydrated and coordinated by the guanines (eight coordination), whereas our DNAzyme is likely only partially coordinated. Our previous mutation studies also indicated that the DNAzyme did not form a G4 structure (45). Such low coordination number may also allow easy conversion between the Na^+^ and K^+^ binding states, consistent with our data above. When Na^+^ and K^+^ are compared for Ce13p binding, K^+^ has a larger change in Δ*H* and Δ*S*, suggesting that it has more coordination sites with the DNAzyme. Therefore, this experiment provided interesting thermodynamic insights that are consistent with the property of the DNAzyme.

### Probing the enzyme loop

**Figure 6.**
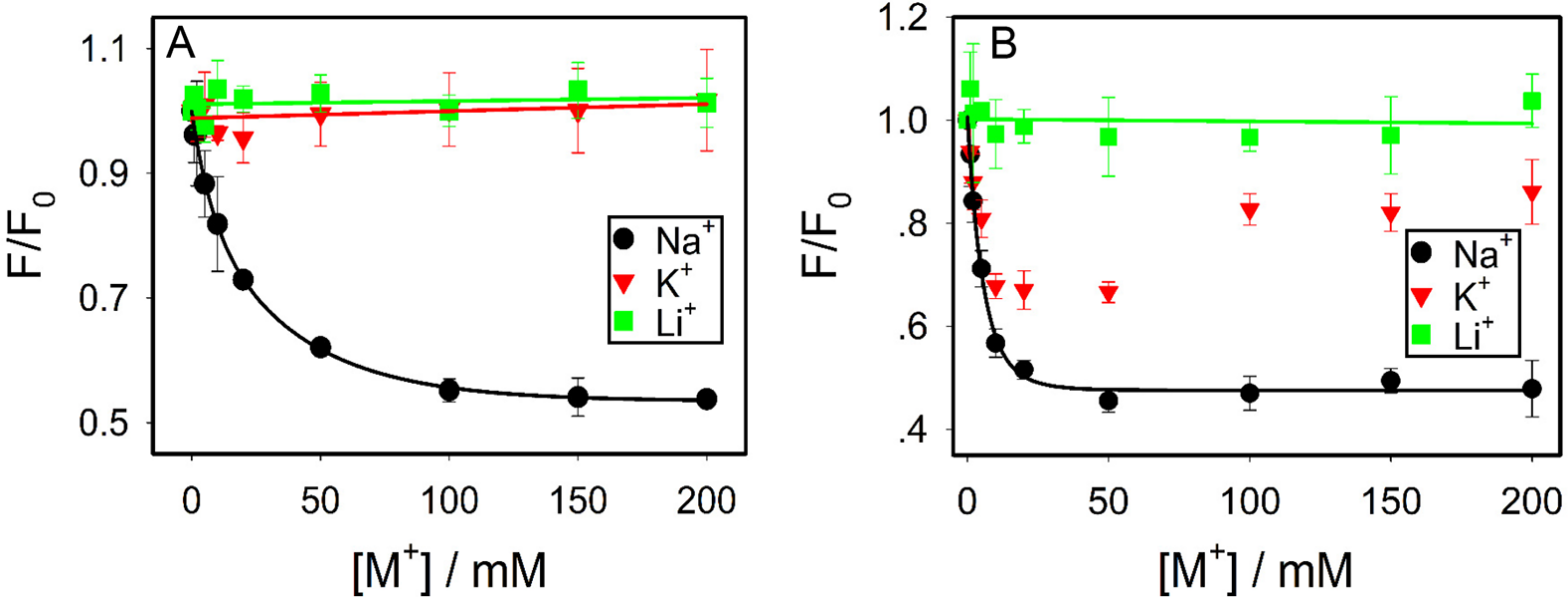
Probing the enzyme loop of Ce13d by labeling a 2AP at the A8 position at (**A**) 25°C and (**B**) 7°C. In (B), K^+^ showed a two-stage binding with the second stage started after ∼10 mM K^+^.

All of the above work labeled the 2AP in the substrate strand, while the main component of the aptamer is in the enzyme loop. Therefore, probing the enzyme loop may offer complementary information. Based on Ce13d, we mutated the A8 adenine to 2AP. At room temperature, we only observed a strong fluorescence change with Na^+^ (Figure 6A) (28). Instead of fluorescence increase, this labeling actually decreased the fluorescence, suggesting more stacking of the 2AP with its neighboring bases. This is consistent with formation of a tight aptamer binding structure.

By lowering the temperature to 7°C, interestingly, we also observed a response to K^+^ (Figure 6B). The pattern of fluorescence change was again different for Na^+^ and K^+^. At low salt concentrations below 10 mM, the fluorescence change by K^+^ and Na^+^ was quite similar. After that, the K^+^ sample showed an increased trend, suggesting a different way of metal binding leading to relaxed base stacking at the 2AP site. By a careful examination of the data for the 2AP labeled in the substrate strand, we also observed two-stage fluorescence change with K^+^ (e.g. Figure 6B, black trace), where fluorescence dropped at high K^+^ concentrations. This is another evidence that K^+^ and Na^+^ were bound differently by the DNAzyme.

Considering these findings together, we now have more complete picture. At low K^+^ concentration (e.g. below ∼10 mM), its binding is similar to that of Na^+^. This can explain that K^+^ has a weak but reproducible cleavage activity. After that, more K^+^ was bound in a different way compared to Na^+^ (can be interpreted as misfolding the DNAzyme), and the cleavage activity decreased with further increase of K^+^ concentration.

### Probing the enzyme by DMS footprinting

**Figure 7.**
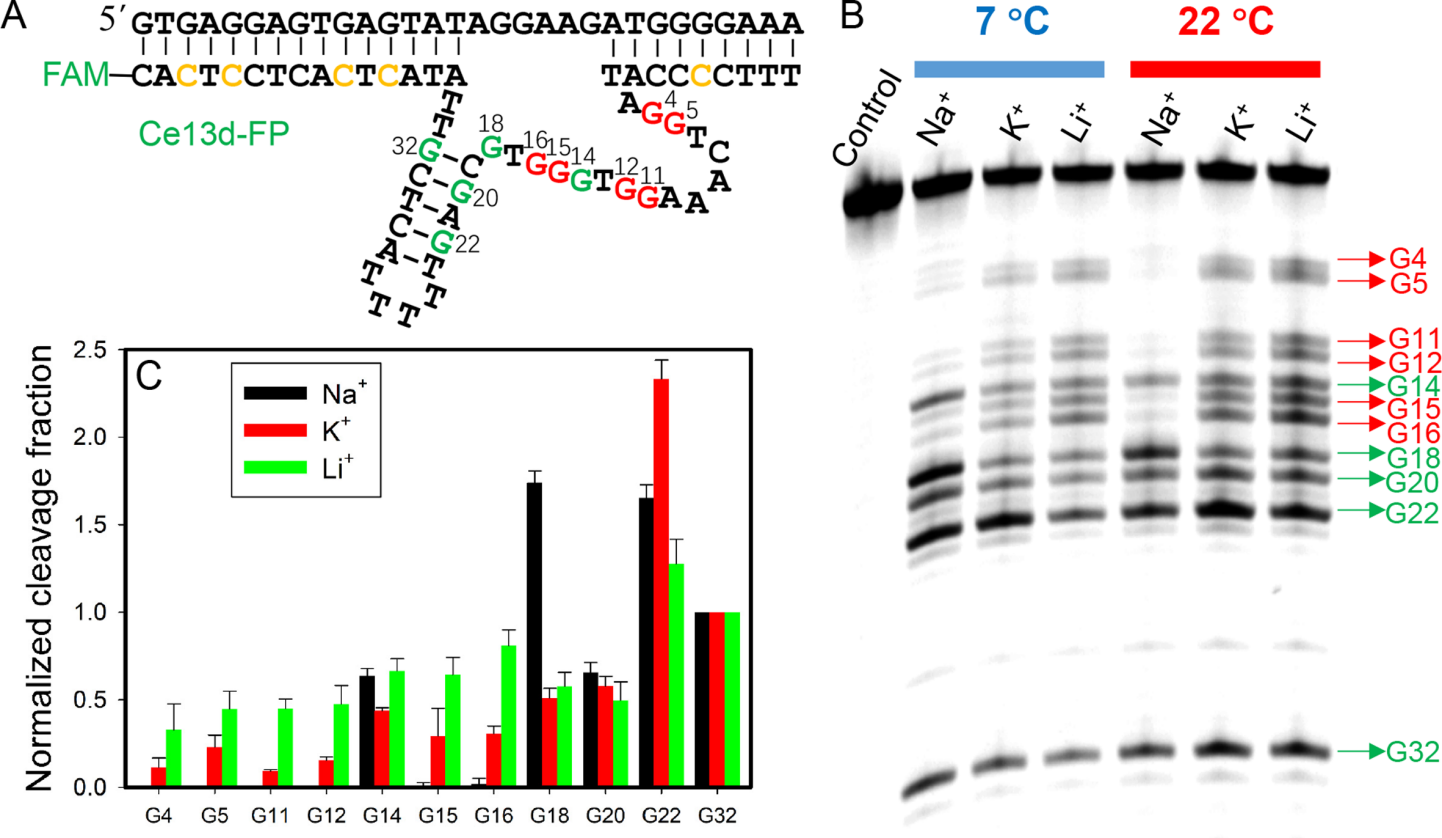
(**A**) The DNAzyme sequence used for DMS footprinting. The FAM label was on the enzyme strand, and the guanines in the substrate binding arm of the enzyme strand were replaced by cytosines. (**B**) A gel micrograph with 600 mM various metal ions. The DMS incubation time was longer at 7°C to achieve sufficient methylation. (**C**) Quantification of the normalized cleavage fraction using G32 as a reference for normalization at 7°C. Na^+^ protected most of the guanines in the enzyme loop, while K^+^ had more protection than Li^+^.

Most of the above studies relied on 2AP fluorescence. To further understand metal binding, we also employed DMS footprinting for an independent verification (25). DMS can methylate guanines and the methylated guanines can be cleaved by piperidine. Folded DNA may protect certain guanines from DMS and these positions may be less methylated. We labeled the enzyme strand with a FAM and replaced the guanines in its substrate binding arms by cytosines to simplify data analysis. The corresponding bases in the substrate strand (non-cleavable) were also changed to keep base pairing (Figure 7A). The DNAzyme complex was respectively incubated with 600 mM Li^+^, Na^+^ and K^+^ at 22°C or 7°C for footprinting. A representative gel micrograph is shown in Figure 7B, where each band corresponded to a guanine in the enzyme strand (see assignment on the right). By a simple visual observation, the cleavage pattern was very different for Na^+^, since many bands completely disappeared indicating folding of the enzyme loop into a compact structure to resist methylation. This is consistent with the presence of a Na^+^ aptamer. The patterns of K^+^ and Li^+^ were also different. To have a quantitative understanding, we measured the band intensity using the G32 as a reference in each lane (Figure 7C). Relative to G32, all the guanines from G4 to G16 (except for G14) showed nearly no cleavage with Na^+^, and most cleavage with Li^+^. In other words, K^+^ protected the guanines more effectively than Li^+^ did, and the relative protection at each guanine was also different for K^+^. Therefore, Li^+^, Na^+^ and K^+^ also had different relative cleavage yields in different positions, supporting the notion that the DNAzyme was folded in different ways in the presence of these three metals. In particular, K^+^ also showed certain protection but its pattern was quite different from that of Na^+^, which is consistent with the conclusion from our 2AP data.

### Salt concentration and cleavage activity

Based on the above data, Li^+^, Na^+^ and K^+^ are three different types of metals for interacting with the DNAzyme. Na^+^ can bind to the aptamer loop in a way to promote the cleavage activity. K^+^ can also bind to the DNAzyme but in a different way and this binding can inhibit the activity (can be interpreted as misfolding). Li^+^, on the other hand, cannot specifically bind the DNAzyme in a way to change the local base stacking of the 2AP at two labeled positions. Li^+^ can only bind via non-specific electrostatic interactions.

**Figure 8.**
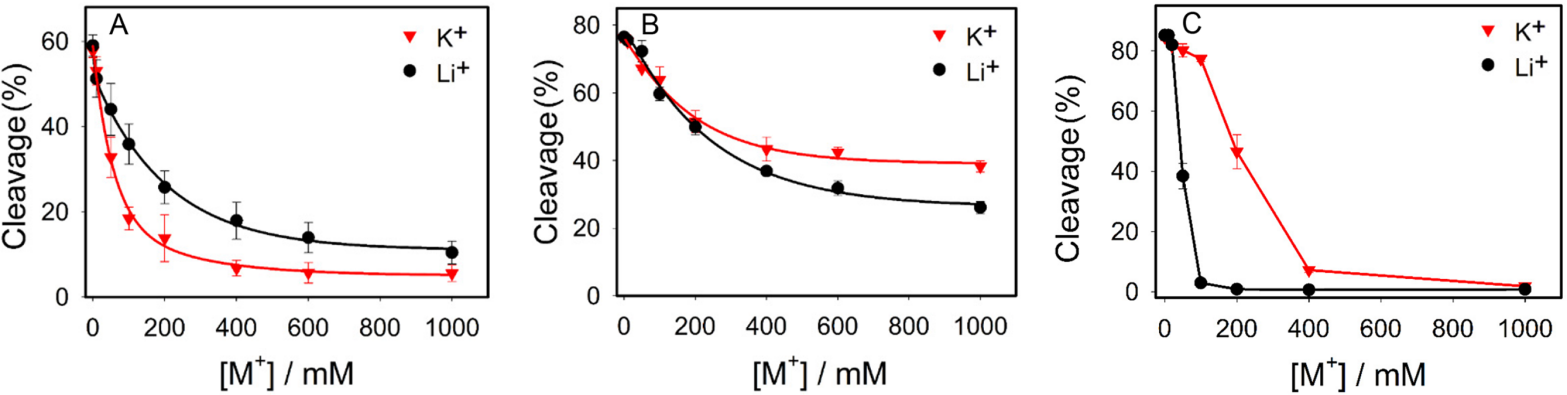
Inhibition of the cleavage activity of (**A**) NaA43T DNAzyme with 25 mM Na^+^, (**B**) 17E DNAzyme with 2 mM Mg^2+^ and (**C**) Tm7 DNAzyme with 10 μM Er^3+^ by different concentrations of Li^+^ and K^+^ at 25°C.

In general, metal-dependent DNAzymes are inhibited by a high ionic strength (38), since salt can screen the interaction between the metal cofactor and DNAzyme. In this work, Na^+^ is the metal cofactor, while Li^+^ and K^+^ might serve to screen the interaction between Na^+^ and the DNAzyme. We wanted to test if inhibition by Li^+^ and K^+^ was different. We measured the cleavage activity of NaA43T with 25 mM Na^+^ in the presence of different concentrations of Li^+^ or K^+^ (Figure 8A). As expected, both Li^+^ and K^+^ inhibited the cleavage activity in a concentration-dependent manner. Interestingly, K^+^ inhibited the activity more than Li^+^ did. From a pure electrostatic standpoint, we would expect Li^+^ to be a more potent inhibitor due to its higher charge density. For example, Li^+^ can increase the melting temperature of DNA more than Na^+^ or K^+^ (46).

**Figure 9.**
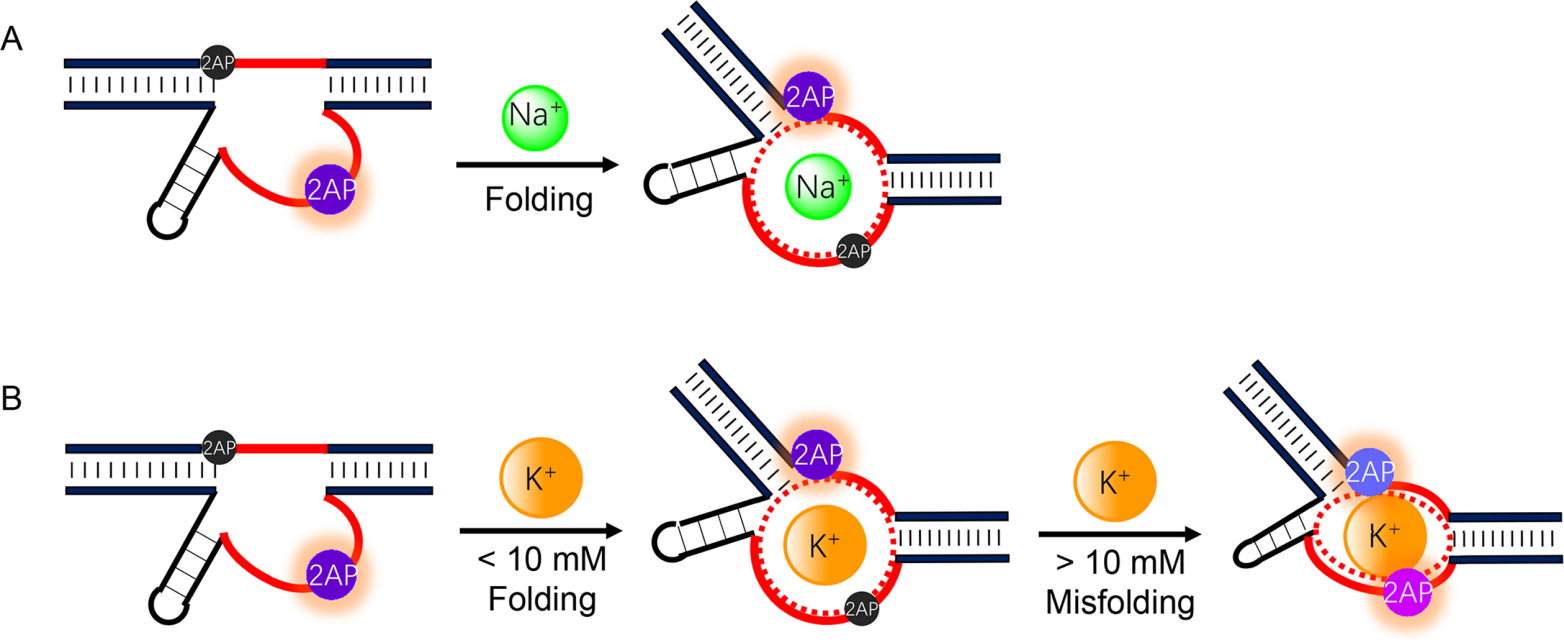
Schemes of metal binding by the DNAzyme in the presence of (**A**) Na^+^ and (**B**) K^+^. Two 2AP fluorophores are labeled for describing the metal binding process, although our experiments were performed only with singly labeled DNAzymes.

To confirm this, we repeated this reaction using two other DNAzymes: 17E (see Figure 1E for structure) with Mg^2+^ (Figure 8B); and Tm7 with Er^3+^ (Figure 8C). For these two DNAzymes, Li^+^ indeed was a stronger inhibitor. This result suggested that the stronger inhibition of NaA43T by K^+^ was likely due to its specific binding to the DNAzyme and thus inhibited the activity more than the non-specific Li^+^ ions. K^+^ binding created an additional barrier for Na^+^ binding. Taking all the data together, we summarized our findings in Figure 9. Ce13d and NaA43 are unique DNAzymes since their specificity for Na^+^ was from an aptamer. This was only seen in another DNAzyme that is specific for Ag^+^ (38,47), while most DNAzymes use their metal cofactors to interact with the scissile phosphate and no aptamers were identified (30). Therefore, this study also complements to the metal binding studies using other DNAzymes (48–51).

In this work, we compared Na^+^ and K^+^ for their cleavage activity and binding by a DNAzyme. Metal binding was probed using 2AP fluorescence and DMS footprinting. Both Na^+^ and K^+^ could bind to the DNAzyme, and at 7°C the saturated fluorescence difference was only ∼4-fold, while the *K*_d_’s werer nearly identical for these two metals. From cleavage activity, however, the selectivity for Na^+^ over K^+^ was much higher, ranging from a few hundred to a few thousand fold. Therefore, catalysis can amplify the difference in metal binding in this case, and this increased difference was rationalized by misfolding. The way of DNAzyme binding to K^+^ was different from its binding to Na^+^, and this enabled the excellent selectivity for Na^+^ in terms of catalytic activity.

This mechanism has not been previously reported for sensing of sodium or potassium by biomolecules. For example, size selectivity is the mechanism for discriminating K^+^ from Na^+^ in potassium channels. The cations are largely dehydrated to cross the channel and the channel pore size fits nicely for K^+^ but not Na^+^ (52), although the exact mechanism of specificity is still under debate. The structure of Na^+^ channels has also been studied and its small size only allows Na^+^ ions to pass while inhibiting K^+^ (53). Aside from the core region of the channel, the channel itself has a lot of other parts to ensure very high specificity. For simple crown ethers, the size-dependent recognition also have only a few to less than 100-fold of selectivity for their target metals (54). Therefore, our DNAzyme compares very favorably with these recognition mechanisms.

For G4 DNAs, their overall structures are more flexible compared to those protein channels since they contain only a small DNA unit and thus are more likely to accommodate cations of different sizes. In the DNAzymes, the strategy is completely different. In nature, specific folding (or conformational change) is an important way to achieve signaling in cells (55). Here, we clearly articulated an interesting mechanism to distinguish Na^+^ from K^+^, showing much better selectivity than protein and other DNA-based mechanisms.

## CONCLUSION

In summary, we used a highly sensitive 2AP-based double mutant Ce13p DNAzyme and low temperature to boost the binding and the fluorescence signal for both Na^+^ and K^+^. This allowed us to systematically compare metal binding and catalytic activity of these two metals. K^+^ was found to have a low, yet reproducible cleavage activity at 10 mM (454-fold lower than that of Na^+^), while further increase of metal concentration inhibited the K^+^ activity, but boosted the Na^+^ activity. This difference was attributed to a misfolded state by K^+^. Binding of both Na^+^ and K^+^ by the DNAzyme was specific since single base mutation could inhibit the signal for both metals. A typical G4 aptamer can only discriminate binding of these two metals by <50-fold, and indeed based on just the 2AP fluorescence, one cannot effectively distinguish Na^+^ and K^+^ using the DNAzyme. The way for the DNAzyme to achieve even higher discrimination between Na^+^ from K^+^ is to misfold the DNAzyme in the presence of K^+^, which inhibited the catalysis. Metal binding takes place at physiologically relevant concentrations of these metals and such strategies might be of biological relevance in terms of discriminating these metals. This work has broadened our fundamental understanding of the nucleic acids in terms of binding metal ions.

## Supplementary Material

Supplementary Data
