# Supplementary material for: Misfolding of a DNAzyme for ultrahigh sodium selectivity over potassium
Source: Nucleic Acids Res. 2018 Sep 12;46(19):10262–71. doi: 10.1093/nar/gky807 (PMC6212836; doi:10.1093/nar/gky807)
Supplement: Supplementary Data [file gky807_supplemental_files.docx]

**Misfolding of a DNAzyme for ultrahigh sodium selectivity over potassium**

Yanping He^1,^^2^, Da Chen^1^*, Po-Jung Jimmy Huang^2^, Yibo Zhou^3^, Lingzi Ma^2^, Kexin Xu^1^, Ronghua Yang^3^, and Juewen Liu^2^*

1. State Key Laboratory of Precision Measurement Technology and Instruments, University of Tianjin, Tianjin, 300072, China

Email: dachen@tju.edu.cn

2. Department of Chemistry, Waterloo Institute for Nanotechnology, University of Waterloo, Waterloo, Ontario, N2L 3G1, Canada

Email: liujw@uwaterloo.ca

Fax (+1) 519-746-0435

Phone: (+1) 519-888-4567 extension 38919

3. School of Chemistry and Biological Engineering, Changsha University of Science and Technology, Changsha 410114, P. R. China

**Table S1**. DNA sequence used in this work. rA= ribo-adenine; FAM= carboxyfluorescein; [2AP]=2-aminopurine.

| **DNA names** | **Sequences and modifications (from the 5'-end)** |
| --- | --- |
| Ce13d | TTTCGCCATAGGTCAAAGGTGGGTGCGAGTTTTTACTCGTTATAGTGACTCGTGAC |
| Ce13p | TTTCGCCATAGGTCAACGGTGGGTGCGAGTTTTTACTCGTAATAGTGACTCGTGAC |
| NaA43T | TTTCGCCATAGGTCAAAGGTGGGTGCGAGTTTTTACTCGGCGGTTAGTGACTCGTGAC |
| G4 | TTTCGCCATGGGTTAGGGTTAGGGTTAGGGTATAGTGACTCGTGAC |
| Tm7 | TTTCGCCATCTTCGATACTCTCTTTGACTCGTGAC |
| 17E | TTTCGCCATCTTCTCCGAGCCGGTCGAAATAGTGACTCGTGAC |
| Ce13p-G_4_T | TTTCGCCATATGTCAACGGTGGGTGCGAGTTTTTACTCGTAATAGTGACTCGTGAC |
| Ce13p-A_9_T | TTTCGCCATAGGTCATCGGTGGGTGCGAGTTTTTACTCGTAATAGTGACTCGTGAC |
| Ce13p-G_11_T | TTTCGCCATAGGTCAACTGTGGGTGCGAGTTTTTACTCGTAATAGTGACTCGTGAC |
| Ce13d-G_4_T | TTTCGCCATATGTCAAAGGTGGGTGCGAGTTTTTACTCGTTATAGTGACTCGTGAC |
| Ce13d-A_9_T | TTTCGCCATAGGTCATAGGTGGGTGCGAGTTTTTACTCGTTATAGTGACTCGTGAC |
| Ce13d-G_11_T | TTTCGCCATAGGTCAAATGTGGGTGCGAGTTTTTACTCGTTATAGTGACTCGTGAC |
| Sub-FAM | GTCACGAGTCACTATrAGGAAGATGGCGAAA-FAM |
| Sub-deoxyribo-2AP | GTCACGAGTCACTAT[2AP]GGAAGATGGCGAAA |
| Ce13d-A_8_2AP | TTTCGCCATAGGTC[2AP]AAGGTGGGTGCGAGTTTTTACTCGTTATAGTGACTCGTGAC |
| Ce13d-FP-FAM | TTTCCCCATAGGTCAAAGGTGGGTGCGAGTTTTTACTCGTTATACTCACTCCTCAC-FAM |
| Sub-dA-FP | GTGAGGAGTGAGTATAGGAAGATGGGGAAA |


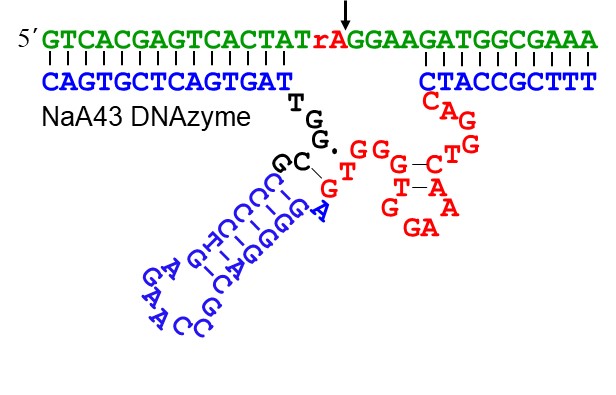


**Figure S1**. The secondary structure of the NaA43 DNAzyme reported in the original paper by Lu and coworkers S([1](#_ENREF_1)). In the current work, we have shorted it mainly in the hairpin region to make the NaA43T DNAzyme, so that it can be more directly compared with the Ce13d DNAzyme.


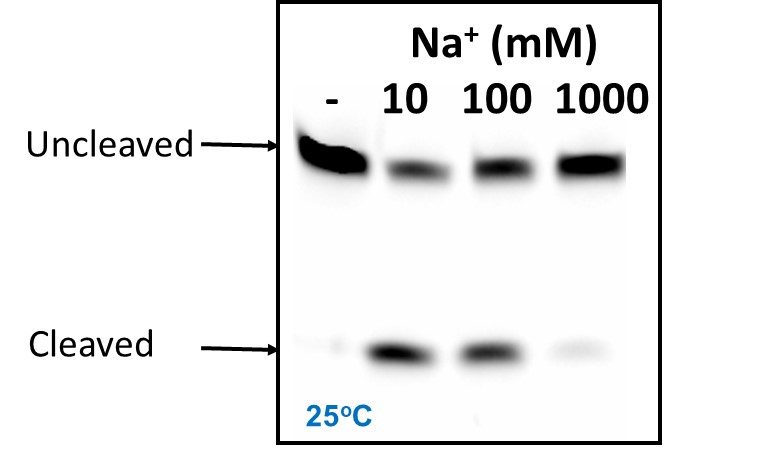


**Figure S2**. The Ce13d cleavage activity tested in different concentrations of Na^+^ containing 10 µM Ce^3+^ at room temperature in buffer (50 mM MOPS, pH 7.0) for 60 min. Although Na^+^ was required for its activity, higher Na^+^ concentration fully inhibited the activity. For this reason, we used the NaA43T DNAzyme in this work for activity measurement, which required only Na^+^ but not Ce^3+^.


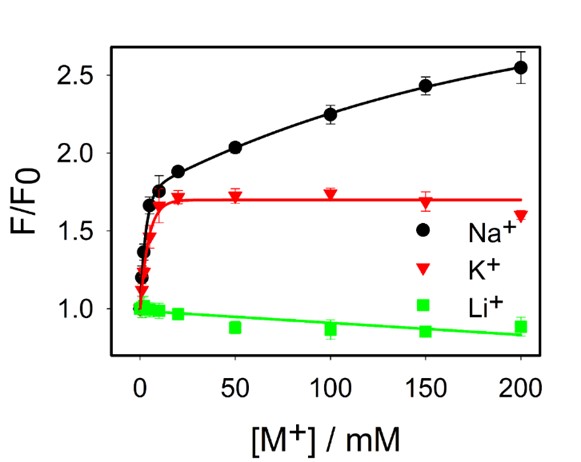


**Figure S3**. The 2AP fluorescence for the original Ce13d DNAzyme, and the 2AP was labeled in the substrate strand. The fluorescence was monitored in the presence of increasing concentrations of Na^+^, K^+^ or Li^+^ at 7 ^o^C in buffer (50 mM HEPES, pH 7.4, 25 mM LiCl).


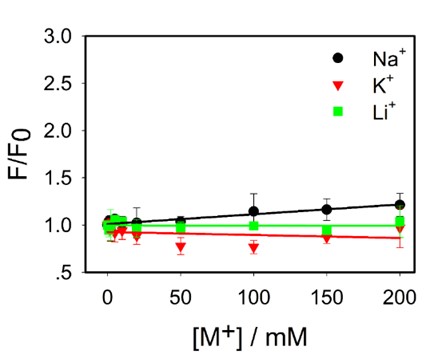


**Figure S4**. Titration of the three metals to the 2AP-labeled NaA43T at 7 ^o^C in buffer (50 mM HEPES, pH 7.4, 25 mM LiCl). The 2AP labeled substrate strand was used. Only a very moderate increase in the Na^+^ fluorescence was observed.


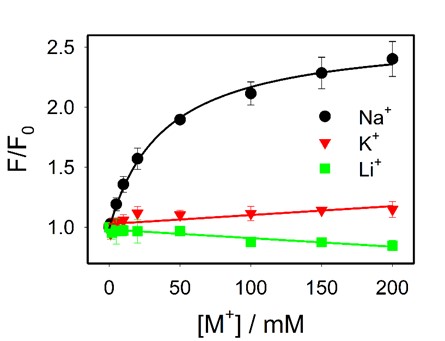


**Figure S5**. Titration of the three metals to the wild-type Ce13d DNAzyme with the 2AP labeled at the substrate strand at room temperature in buffer (50 mM HEPES, pH 7.4, 25 mM LiCl). The change of signal was very moderate and reached only 2.5-fold for Na^+^.


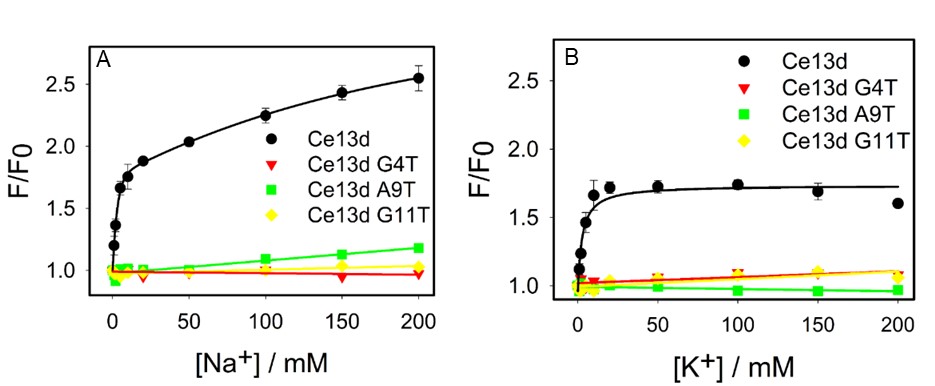


**Figure S6**. Titration of (A) Na^+^ and (B) K^+^ to the DNAzyme complex with either Ce13d or its mutant at 7 ^o^C in buffer (50 mM HEPES, pH 7.4, 25 mM LiCl). In these case, the same 2AP labeled substrate strand was used. We still observed that a single mutation in the enzyme strand has nearly fully inhibited Na^+^ or K^+^ binding, confirming specific folding and specific misfolding.

**Additional references**

S1. Torabi, S.-F., Wu, P., McGhee, C.E., Chen, L., Hwang, K., Zheng, N., Cheng, J. and Lu, Y. (2015) In vitro selection of a sodium-specific DNAzyme and its application in intracellular sensing. *Proc. Natl. Acad. Sci. U.S.A.*, **112**, 5903-5908.
